# Health-Enhancing Physical Activity (HEPA) and Its Barriers Among Working Women at Mansoura University, Egypt

**DOI:** 10.3390/diseases12120318

**Published:** 2024-12-07

**Authors:** Wael Ramadan, Mariam Abu Alim, Mohammed Khamis, Abdel-Hady El-Gilany

**Affiliations:** 1 Department of Sports Training, Faculty of Physical Education, Mansoura University, Mansoura 35516, Egypt; 2 Department of Sport Science, Yarmouk University, Irbid 21163, Jordan; mariam.a@yu.edu.jo; 3 Department of Sports Recreation, Mansoura University, Mansoura 35516, Egypt; drmohamedkhamis1980@gmail.com; 4 Department of Public Health, Faculty of Medicine, Mansoura University, Mansoura 35516, Egypt; ahgilany@gmail.com

**Keywords:** physical activity, barriers, women

## Abstract

Background: Identifying physical activity (PA) and its barriers among middle-aged women may aid in the development of age-specific health promotion interventions. In Egypt, women, particularly those in the workforce, typically face numerous barriers that result in low levels of PA. This research seeks to assess the prevalence of health-enhancing physical activity (HEPA) among working women at Mansoura University and identify the associated barriers. Methods: This cross-sectional study employs a quantitative methodology that includes an analytic component. We studied a non-random sample of 760 Egyptian women employed at Mansoura University. Sociodemographic data were collected, and the international physical activity questionnaire (IPAQ) short form was used to assess the level of PA. Additionally, the Barriers to Being Active Quiz (BBAQ) was used to assess barriers to PA. Results: One-fourth of women engage in HEPA; 44.9% are classified as minimally active, while 30.1% are inactive. Multivariable logistic regression analysis showed the type of job is an independent predictor of HEPA, with ARR of 1.7 for manager and administrative roles. The total BBAQ score significantly predicts HEPA (ARR = 0.94). Social influences and lack of willpower scores are significant independent predictors of HEPA (ARR = 0.9 and 0.8, respectively). Most individuals encounter two to five barriers, with only 11.3% experiencing no PA barriers. The most frequently reported barrier is the lack of energy (80.4%), followed by the lack of resources, willpower, and time (74.04%, 69.6%, and 69.4%, respectively). Conclusions: The prevalence of HEPA is low among working women at Mansoura University. Policymakers can utilize findings to promote engagement with and adherence to physical activity.

## 1. Introduction

Women’s obligations and work schedules are complex and carry substantial responsibilities, varying considerably due to social, cultural, and individual factors, including parenting, household and family care, and professional duties. Balancing family, work, and personal life entails considerable responsibility and presents numerous challenges, particularly as women frequently encounter societal pressures to succeed in both domestic and professional domains. The disproportionate burden of responsibilities on women significantly affects their overall quality of life, particularly in relation to self-care and mental health. Research indicates that women experience greater guilt than men regarding the prioritization of self-care, which is identified as a significant barrier to participation in PA and sports after work hours [1].

Physical activities confer health benefits and necessitate energy expenditure, which occurs during daily activities such as work, transportation, and leisure, provided these activities are performed at moderate to vigorous intensity [2]. The World Health Organization (WHO) reports that fewer than 40% of individuals adhere to the recommended physical activity (PA) guidelines. Consequently, they may not experience the advantages linked to engaging in healthy PA. Research indicates that most individuals do not achieve the recommended levels of PA, primarily due to sedentary behaviors associated with daily routines, including workplace environments [3]. The impact of a sedentary work environment, as evidenced by policies, flexibility, and culture, is crucial to employees’ PA levels and overall health [4]. Research indicates that during extended working hours, over 75% are is allocated to sedentary behaviors, which can account for approximately 60–70% of employees’ waking hours daily [5,6,7].

Therefore, numerous interventions have aimed at reducing physical inactivity through the efforts of policymakers, public health sectors, and researchers by promoting workplace leisure activities, addressing challenges, and implementing health-related PA interventions [8].

There are increased concerns regarding women’s wellbeing in Egypt, particularly related to their working hours, alongside a scarcity of literature evaluating the barriers and facilitators that influence employees’ engagement in physical activities within or outside the workplace. Researchers have been encouraged to identify barriers encountered which may reduce absenteeism and promote health and wellbeing-related PA interventions specifically aimed at female employees. This study aims to assess the level of PA and identify barriers to promoting healthy physical activities and factors associated with HEPA among working women at Mansoura University, Egypt.

## 2. Materials and Methods

Study design and duration: This is a cross-sectional study with an analytic component, utilizing a quantitative approach, conducted from January to February 2024.

Target population: Egyptian women working in Mansoura University without any disability. Working was operationally defined as permanent official work for cash (i.e., a paid job), whether working as academic or administrative staff. All of them are working for 8 h daily according to the Egyptian labor laws. Students were not our target population.

Sample size calculation: Sample size calculation was performed utilizing Medcalc version 15.8 (https://www.medcalc.org/ (accessed on 15 January 2024)). The main focus of this study is HEPA prevalence among employed women. A pilot study involving 50 working women who were not part of the full-scale study indicated an HEPA prevalence of 26.0%. Considering a 5% alpha error, 90% study power, and 5% precision, the required sample size was a minimum of 741 participants. In total, 760 employed women participated in the survey.

Data collection approach: The data collection approach involved the use of online methods, specifically utilizing Google Forms distributed through emails, WhatsApp, and other platforms. Thus, a convenience non-random sample of women was included, and the characteristics of the study population may not be similar to the target population in general.

The Arabic questionnaire comprised sociodemographic information (marital status, education, age, and occupation), self-reported chronic diseases, a quiz on PA barriers, and the International Physical Activity Questionnaire (IPAQ) short form [9].

The IPAQ short form was utilized to evaluate PA levels [10]. The Arabic version can be accessed at Garashi et al. (2020). It is a valid and reliable measure for physical activity [11]. The short Arabic version was recognized to be valid and reliable to assess PA level [12,13].

The assessment encompasses PA in four domains: leisure time, domestic and gardening tasks, work-related activities, and transport-related movements. The instrument assesses three specific types of activity, vigorous-intensity, walking, and moderate-intensity, utilizing a seven-item scale that yields distinct scores for each category of activity. The total score is calculated by summing the duration (in minutes) and frequency (in days) of walking, moderate-intensity, and vigorous-intensity activities. PA was categorized into three levels: low or physically inactive, moderate, and high or HEPA. The activity was calculated by weighting each type according to its energy expenditure, defined in metabolic equivalents (METs), which are multiples of the resting metabolic rate, resulting in a score expressed in MET minutes. A MET minute is calculated by multiplying the MET score by the duration in minutes of the activity performed. MET minute scores correspond to kilocalories for an individual weighing 60 kg. Kilocalories can be calculated from MET minutes using the following equation: MET-min × (weight in kilograms/60 kg). The determined MET values were obtained from the mean MET score corresponding to each activity type. The PA score was calculated as follows: walking = 3.3 METs, moderate physical activity = 4.0 METs, and vigorous PA = 8.0 METs. The PA was classified into three levels: physically inactive, HEPA, and minimally active [9].

The following three levels of PA are suggested for the categorical score:

1. Inactive:
  Either of the following criteria:
  ▪ No reported activity;
  ▪ Inadequate activity is reported to satisfy the criteria for categories 2 or 3.
2. Minimally Active:
  Any one of these three criteria:
  ▪ Three or more days of vigorous activity lasting not less than 20 min each day;
  ▪ Five or more days of walking or moderate-intensity activity for a minimum of 30 min daily;
  ▪ Engaging in five or more days of a combination of walking and moderate-to-vigorous-intensity activities, accomplishing at least 600 MET-min/week.
3. HEPA active:
  Any one of the following two criteria:
  ▪ Vigorous-intensity activity should be performed on a minimum of three days, resulting in an accumulation of at least 1500 MET minutes per week;
  ▪ Seven or more days of any combination of vigorous-, moderate-intensity, or walking activities accomplishing a minimum of 3000 MET minutes per week [9].

The Barriers to Being Active Quiz (BBAQ) was developed by CDC and it comprises 21 items to be assessed on a Likert scale ranging from 0 to 3. It was translated into Arabic and validated in a previous study in Saudi Arabia (Al Salim, 2023) [14]. These barriers can be classified into seven categories: lack of resources, skill, injury, motivation, energy, social influences, and time. Respondents rate the degree of activity interference on a 4-point scale, ranging from 0 = “very unlikely” to 3 = “very likely”. Each category comprises three items, totaling 9 as a maximum score. A category containing five or more is regarded as a significant barrier that must be addressed [15,16].

The BBAQ appears to be a valid and reliable tool for assessing PA barriers. The BBAQ test, comprising 21 items, yielded a coefficient alpha score of 0.87, signifying strong internal consistency reliability. Factor analysis was performed to validate the BBAQ. All seven categories exhibited a range of loading between 0.87 and 0.64. Consequently, the BBAQ appears to assess a single construct related to PA barriers [14]. Our study revealed that Cronbach’s alpha is satisfactory for the total scale (0.88) and its domains viz. lack of time (0.77), social influence (0.65), lack of energy (0.7), lack of willpower (0.68), fear of injury (0.65), lack of skill (0.69), and lack of resources (0.75).

Data analysis: The 17.0 V of the SPSS software was utilized for data analysis (SPSS Inc.-Chicago-IL-USA). Descriptive statistics were calculated for all variables (qualitative) that were presented as frequencies and percentages. The chi-squared test was used to compare categorical data. The crude risk ratios (CRRs) and their 95% CIs were calculated. All were entered into two multivariate binary logistic regression models using the enter method to detect the independent predictors of HEPA. Adjusted risk ratios (ARRs) and their 95% CIs were calculated. Internal consistency of the BBAQ and its domains were measured by Cronbach’s alpha. Also, confirmatory factor analysis for BBAQ was conducted in our study population. The statistical significance level was set at ≤0.05.

## 3. Results

Table 1 shows that most women were aged 30 years and older, with a mean of 34.5 ± 7.9. About two-thirds (63.7%) held postgraduate degrees, 48.4 were single, and 57.8% had self-reported a chronic disease.

Overall, one-fourth of women were HEPA, and 44.9% and 30.1% were minimally active and inactive, respectively. The majority of them had two to five PA barriers, and only 11.3% had no barriers. Lack of energy was the most frequently reported barrier (80.4), followed by lack of resources, lack of willpower, and lack of time (74.04%, 69.6%, and 69.4%, respectively (Table 2).

Table 3 indicates that HEPA was significantly reduced in women aged ≤30 years (CRR = 0.9, 95% CI 0.8–0.98). Nevertheless, it was notably higher in managers/administrative personnel and others when compared to university staff (CRR = 1.5, 95% CI 1.1–2.0 and 1.55, 95% CI 1.1–2.2, respectively). The trend indicates a reduction in HEPA as the number of barriers increases, with CRR values of 0.6 for three and 0.3 for six barriers relative to the absence of barriers. Various barriers were linked to a low CRR, falling between 0.8 and 0.9.

Table 4 shows that type of job is an independent predictor of HEPA in both models, with ARR values of 1.7 for manager and administrative roles. The total BBAQ score significantly predicts HEPA in model 2 (ARR = 0.94). In model 1, social influences and lack of willpower scores are significant independent predictors of HEPA (ARR = 0.9 and 0.8, respectively).

## 4. Discussion

Sedentary lifestyle and high levels of inactivity represent a significant global public health concern. Despite the implementation of the Global Strategy on Physical Activity and Health (WHO 2003), rates remain notably high, particularly among women [7]. Lifestyle with regard to obligations and the overall socioeconomic status of women play an integral role in their HEPA. As the previous literature addressed, most barriers affecting women’s HEPA are increasing with age [17], which is what the results of this study showed. A prevalent explanation for this phenomenon is the heightened family responsibilities and diminished leisure time experienced by this age group. In Egyptian culture, women face extended family obligations and responsibilities, which diminishes their energy and willpower throughout the day [18]. A review study by Chaabane et al. (2021) examined the barriers and facilitators related to physical activity in the Middle East and North Africa region. The findings indicated that sociodemographic factors, particularly being married and of advanced age, negatively impacted female participation in PA. This systematic review indicated that one of the most prevalent barriers to engaging in PA in the region was gender and cultural norms [19].

This study’s sample also examined the barriers faced by working women at Mansoura University, adding an additional layer of barriers related to HEPA, specifically the issue of long working hours, as noted in the previous literature. The current study revealed that the total score of barriers (model 2) and scores of social influences and lack of wellbeing domains (model 1) rather than their type is most important in predicting HEPA. Furthermore, some barriers can be changed and others cannot. We dealt with barriers as equal in their contribution to HEPA. This is not the case in real life. However, it is not clear whether there is an additive effect or multiplicative synergistic effects of these barriers. The results of this study indicate that a lack of time was prevalent, although the sample size for the under-30 age group was smaller. Comparable findings were noted in the review by Santos and Miragaia (2023) and Baek et al. (2023); employment exceeding 40 h per week was significantly associated with physical inactivity and a reduced probability of participating in high PA and HEPA levels [3,20].

The availability of recreational areas and gyms may contribute to the identified barrier of resource scarcity. A study by Mariam and Mazin (2019) [21] examined the barriers associated with long working hours for women employed at universities. They addressed the lack of resources resulting from limited gym availability for women and insufficient suitable sports facilities. Operating hours for women in most gyms typically range from early morning to evening, making it challenging for those with long working hours to enroll. Conversely, their feelings of guilt regarding the time spent away from children and personal relationships impact their willpower. Alrimali (2023) identified similar findings, noting that the primary PA barriers were insufficient resources and lack of willpower. Additionally, psychological factors such as self-efficacy and willpower are significantly correlated with engaging in PA [22].

The findings of this study enhance the understanding of the barriers that hindered participants from engaging in or benefiting from PA, which aims to improve health, as demonstrated by the calculated regression model.

The sample exhibited a HEPA prevalence of 25%. This suggests that 25% of the participants partake in health-beneficial PA. This prevalence aligns with multiple studies that indicate differing rates of PA adherence across various populations. An increasing number of barriers is associated with a decreasing prevalence of HEPA. The statistical analysis indicated that a greater number of barriers correlated with a reduced prevalence of HEPA. Individuals encountering greater obstacles, including time constraints, social influences, or limited resources, were less likely to participate in HEPA. This finding highlights the necessity of addressing various barriers to promoting PA. Different types of barriers exert distinct effects on HEPA prevalence. The constraints of insufficient time, energy, and resources were significantly correlated with reduced PA levels. Targeted interventions aimed at addressing these specific barriers may be essential for enhancing PA rates [23].

The study’s bivariate analysis revealed a significant association between HEPA and age as a categorical variable. Younger participants (<30 years) showed a higher prevalence of HEPA (31.2%) compared to older participants (≥30 years) at 22.6%. This finding aligns with the standing literature, signifying that younger individuals are usually more active. This is potentially due to higher levels of motivation, greater physical capacity, and lifestyle aspects [24]. However, including age as a continuous variable in logistic regression was found to be a non-significant predictor of HEPA. This may have been due to the strong effects of confounders other than age in this relatively humongous sample of younger-age working women. This contradiction needs to be addressed by future large-scale studies including women in different occupations. The level of education did not have any statistical significance between those with college/secondary education and those with postgraduate degrees, and their prevalence of HEPA was not high. This result suggests that higher education levels do not necessarily correlate with increased HEPA, which contradicts some research studies that associate higher education with greater health awareness and activity levels. HEPA prevalence exhibited significant variation based on job type. The prevalence among university staff was 18.5%, which is lower than that of managers and administrative staff at 27.3% and others at 28.6%. This variation may be associated with job demands, stress levels, and issues pertaining to work–life balance. Managerial roles may provide greater flexibility for promoting PA compared to university administrative roles [21,25].

The prevalence of HEPA did not differ significantly between those with and without chronic diseases. This result might be attributed to the complex interplay between chronic conditions and physical activity, where individuals with chronic diseases might still engage in PA based on their health status and personal motivation. Also, PA may represent a line of management for some chronic diseases [26,27]. Finally, marital status did not demonstrate any significant impact on the prevalence of HEPA. This finding aligns with studies indicating that marital status is not a significant determinant of PA levels. Nonetheless, the relationship may be affected by additional factors, including children, household duties, and social support [19,22].

Study limitations. The study is exploratory, as it focused solely on working women from a single university and utilized an online setting with a random, convenient number of participants. These factors limit the external validity and generalizability of the findings. Consequently, the results may not apply to women in other occupations, particularly those that demand physical work, such as factories or industries.

## 5. Conclusions

This study has synthesized the body of knowledge on HEPA and its barriers among working women. A significant proportion of women encounter two to five barriers, with only 11.3% reporting the absence of barriers to PA. The primary barrier identified is lack of energy (80.4%), followed by lack of resources (74.04%), lack of time (69.6%), and lack of willpower (69.4%). The type of job is an independent predictor of HEPA in both models, with an ARR value of 1.7 for manager and administrative roles. The total BBAQ score significantly predicts HEPA in model 2 (ARR = 0.94). In model 1, social influences and lack of willpower scores are significant independent predictors of HEPA (ARR = 0.9 and 0.8, respectively). One-fourth of working women engage in HEPA. While 44.9% were minimally active, 30.1% were inactive. Professionals can use findings to promote engagement with and adherence to PA.

## 6. Recommendations

Based on the findings, there is a need to promote HEPA among working women in Mansoura University, especially university staff and those aged 30 years or more, by removing or minimizing different barriers for physical activity. A national large-scale study covering all job categories in different sectors is recommended. It is not clear whether there is an additive effect or multiplicative synergistic effects of multiple barriers on HEPA. This deserves a more comprehensive study.

## Figures and Tables

**Table 1:** Sociodemographic characteristic and medical history of studied women.

	N (%)
Age (Years): <30	208 (27.4)
30 and more	552 (72.8)
Education: College and 2ry *	276 (36.3)
Post-graduate degrees **	484 (63.7)
Job: University staff	227 (29.9)
Mangers and administrative	341 (44.9)
Others ***	192 (25.3)
Marital status: Single	368 (48.4)
Married	281 (37.0)
Widowed/divorced	111 (146)
Chronic diseases (self-reported): No	321 (42.2)
Yes	439 (57.8)

* Only 8 secondary. ** Mainly M.Sc, Ph.D. and Postgraduate Diplomas. *** Physicians, pharmacists, lab. Technicians, engineers, etc., e.g., musculoskeletal, hypertension, diabetes, bronchial asthma, irritable bowel syndrome, etc.

**Table 2:** Prevalence of physical activity and its barriers among studied women.

	N (%)	95% CI
Physical activity levels: Inactive	229 (30.1)	26–33.4
Minimally active	341 (44.9)	41.3–48.4
Health-enhancing (HEPA) activity	190 (25.0)	21.9–28.1
Number of barriers: None	86 (11.3)	9.1–13.6
One	71 (9.3)	7.3–11.4
Two	93 (12.2)	9.9–14.6
Three	125 (16.4)	13.8–19.1
Four	135 (17.8)	15.1–20.5
Five	107 (14.1)	11.6–16.6
Six	89 (11.7)	9.4–14.0
Seven	54 (7.1)	5.3–8.9
Type of barriers *: Lack of time	468 (69.4)	66.0–72.9
Social influences	270 (40.1)	36.4–43.8
Lack of energy	542 (80.4)	77.4–83.4
Lack of willpower	469 (69.6)	66.1–73.1
Fear of injury	188 (27.9)	24.5–31.3
Lack of skills	194 (28.8)	25.4–32.2
Lack of resources	499 (74.04)	70.7–77.4

* Among 689 women with one or more barriers. Categories are not mutually exclusive.

**Table 3:** Bivariate logistic regression analyses of hypothesized predictors of HEPA.

	Total	HEPA N (%)	*p*-Value	ARR (95% CI)
Age (Years): <30	208	65 (31.2)		r(1)
30 and more	552	125 (22.6)	0.02	0.9 (0.8–0.98)
Education: College and 2ry	276	69 (25.0)		r(1)
Post-graduate degrees	484	121 (25.0)	1.0	1.0 (0.9–1.1)
Job: University staff	227	42 (18.5)		r(1)
Mangers and administrative	341	93 (27.3)	0.016	1.5 (1.1–2.0)
Others	192	55 (28.6)	0.014	1.55 (1.1–2.2)
Marital status: Single	368	103 (28.0)		r(1)
Married	281	60 (21.4)	0.053	0.8 (0.6–1.01)
Widowed/divorced	111	27 (24.3)	0.4	0.9 (0.6–1.3)
Chronic diseases (self-reported): No	321	78 (24.3)		r(1)
Yes	439	112 (25.5)	0.7	1.1 (0.8–1.3)
Number of barriers: None	86	40 (46.5)		r(1)
One	71	27 (38.0)	0.3	0.82 (0.6–1.2)
Two	93	23 (24.7)	0.002	0.5 (0.3–0.8)
Three	125	35 (28.0)	0.006	0.6 (0.4–0.9)
Four	135	25 (18.5)	≤0.001	0.4 (0.3–0.6)
Five	107	17 (15.9)	≤0.001	0.3 (0.2–0.6)
Six	89	14 (15.7)	≤0.001	0.3 (0.2–0.6)
Seven	54	16.7 (16.7)	≤0.001	0.4 (0.2–0.7)
Type of barriers *: Lack of time	468	90 (19.2)	≤0.001	0.8 (0.7–0.9)
Social influences	270	39 (14.4)	≤0.001	0.8 (0.7–0.9)
Lack of energy	542	108 (19.9)	≤0.001	0.8 (0.7–0.9)
Lack of willpower	469	89 (19.0)	≤0.001	0.8 (0.7–0.9)
Fear of injury	188	40 (21.3)	0.2	0.9 (−0.86–1.0)
Lack of skills	194	31 (16.0)	≤0.001	0.9 (0.8–0.3)
Lack of resources	499	113 (23.2)	0.1	0.9 (0.85–1.0)

ARR = adjusted relative risk, CI—confidence interval. * Absence of each barrier is the reference category.

**Table 4:** Two models of multivariate logistic regression analysis of predictors of HEPA.

	Model 1			Model 2		
	β	*p*	ARR (95% CI)	β	*p*	ARR (95% CI)
Age (contiuous)	−0.01	0.3	0.99 (0.96–1.01)	−0.01	0.3	0.99 (0.96–1.01)
Job: University staff	-		r(1)	-		r(1)
Mangers and administrative	0.5	0.02	1.7 (1.1–2.6)	0.6	0.01	1.7 (1.1–2.7)
Others	0.5	0.08	1.6 (0.95–2.6)	0.5	0.05	1.6 (1.01–2.7)
Education: College and 2ry	-		r(1)	-		r(1)
Post-graduate degrees	0.2	0.3	1.2 (0.8–1.8)	0.2	0.3	1.2 (0.8–1.7)
Marital status: Single	-		r(1)	-		r(1)
Married	−0.3	0.2	0.4 (0.4–1.2)	−0.4	0.06	0.7 (0.5–1.02)
Widowed/divorced	−0.2	0.2	0.45 (0.3–1.1)	−0.3	0.3	0.8 (0.5–1.3)
Chronic disease: No	-		r(1)	-		r(1)
Yes	0.2	0.3	1.2 (0.9–1.7)	0.2	0.3	1.2 (0.8–1.7)
Number of barriers	0.1	0.5	1.1 (0.9–1.4)	0.1	0.6	1.1 (0.8–1.3)
Total BBAQ score (continuous)				−0.1	0.003	0.94 (0.9–0.98)
Lack of time score (continuous)	−0.1	0.2	0.9 (0.8–1.03)			
Social influences score (continuous)	−0.2	0.003	0.8 (0.7–0.9)			
Lack of energy score (continuous)	−0.1	0.2	0.9 (0.8–1.03)			
Lack of willpower score (continuous)	−0.1	0.03	0.9 (0.8–0.98)			
Fear of injury score (continuous)	0.1	0.3	1.1 (0.96–1.2)			
Lack of skills score (continuous)	−0.1	0.1	0.9 (0.8–1.01)			
Lack of resources score (continuous)	0.1	0.1	1.1 (0.99–1.2)			
Data of model adequacy:						
Model Chi-square	87.7, *p* ≤ 0.001			60.9, *p* ≤ 0.001		
−2log likelihood	770.0			793.9		
Cox and Snell R2	0.11			0.08		
Nagelkerke R@	0.16			0.11		
Hosmer and Lemeshow	15.3, *p* = 0.054			7.8, *p* = 0.45		
Constant	0.3			0.52		
% Correctly predicted	78.3			75.9		
Variables included in the model	Age, education, marital status, job, chronic diseases, lack of time score, social influences score, lack of energy score, lack of willpower score, fear of injury score, lack of skills score, lack of resources score, number of barriers.			Age, education, marital status, job, chronic diseases, total number of barriers, BBAQ score.		

ARR = adjusted relative risk, CI—confidence interval.

## Data Availability

The datasets presented in this article are available from the corresponding author on reasonable request. Due to privacy concerns, some data cannot be publicly shared.
